# Volumetry of Olfactory Structures in Mild Cognitive Impairment and Alzheimer’s Disease: A Systematic Review and a Meta-Analysis

**DOI:** 10.3390/brainsci11081010

**Published:** 2021-07-30

**Authors:** Benoît Jobin, Benjamin Boller, Johannes Frasnelli

**Affiliations:** 1 Department of Psychology, Université du Québec à Trois-Rivières, Trois-Rivieres, QC G8Z 4M3, Canada; benjamin.boller@uqtr.ca; 2 Research Centre of the Institut Universitaire de Gériatrie de Montréal, Montréal, QC H3W 1W5, Canada; 3 Research Centre of the CIUSSS du Nord-de-l’île-de-Montréal, Montréal, QC H4J 1C5, Canada; Johannes.a.frasnelli@uqtr.ca; 4 Department of Anatomy, Université du Québec à Trois-Rivières, Trois-Rivieres, QC G8Z 4M3, Canada

**Keywords:** olfactory bulb, primary olfactory cortex, Alzheimer’s disease, MCI, MRI, meta-analysis

## Abstract

Olfactory decline is an early symptom of Alzheimer’s disease (AD) and is a predictor of conversion from mild cognitive impairment (MCI) to AD. Olfactory decline could reflect AD-related atrophy of structures related to the sense of smell. The aim of this study was to verify whether the presence of a clinical diagnosis of AD or MCI is associated with a volumetric decrease in the olfactory bulbs (OB) and the primary olfactory cortex (POC). We conducted two systematic reviews, one for each region and a meta-analysis. We collected articles from PsychNet, PubMed, Ebsco, and ProQuest databases. Results showed large and heterogeneous effects indicating smaller OB volumes in patients with AD (k = 6, *g* = −1.21, 95% CI [−2.19, −0.44]) and in patients with MCI compared to controls. There is also a trend for smaller POC in patients with AD or MCI compared to controls. Neuroanatomical structures involved in olfactory processing are smaller in AD and these volumetric reductions could be measured as early as the MCI stage.

## 1. Introduction

Alzheimer’s disease (AD) is the main cause of dementia in older adults [1]. According to a large meta-analysis that included 119 studies, the overall point prevalence of dementia due to AD among individuals 60 and older is 40.2 per 1000 persons in community settings [2]. AD is a neurodegenerative pathology characterized by the accumulation of amyloid-β plaques and tau neurofibrillary tangles in the brain that leads to dementia [3]. The neuropathology of AD begins 20 years or more before first cognitive symptoms appear [4]. At the behavioral level, it has been proposed that the first manifestation of AD neuropathology is a complaint of a recent cognitive change known as Subjective Cognitive Decline (SCD) [5] before the manifestation of cognitive deficits known as Mild Cognitive Impairment (MCI) [6]. Although these early clinical stages are useful to better predict who is at risk of developing dementia related to AD, it would be important to find earlier and more specific markers. For instance, only 14% of individuals with SCD and 34% of those with MCI are expected to convert to dementia at least [7,8]. Neurobiological damage related to AD that appears during a silent phase preceding SCD and MCI stages [4,9,10,11] first occurs in the transentorhinal and limbic regions [12,13], which are involved in memory and olfactory processing [14,15,16,17].

The neuropathology of AD is driven by two processes: an extracellular accumulation of amyloid-β proteins (amyloid plaques) and an intracellular accumulation of hyperphosphorylated tau proteins (neurofibrillary tangles) [3,18]. Thal et al. [19] suggest five phases of amyloid-β accumulation: it appears (1) first in the neocortex, (2) then in the allocortex and more precisely the entorhinal region, CA1 region of the hippocampus, and in the insular cortex, (3) then in subcortical nuclei, before involving (4) the brainstem, and (5) the pons and the cerebellum. Concerning tau pathology, Braak and Braak [13] suggest five stages of tau accumulation. First, neurofibrillary tangles are confined to the transentorhinal region and the CA1 region of the hippocampus (stages I-II), then the limbic regions such as the subiculum of the hippocampal formation and the amygdala (stage III–IV), and finally to the isocortex (stages V–VI).

Neurodegeneration can be quantified using magnetic resonance imaging (MRI) which allows in vivo volumetric measurement of neuroanatomic structures. MRI data shows that brain atrophy follows Braak staging and appears first in the medial temporal lobe, in the entorhinal cortex, followed by the hippocampus [20,21,22,23] before progressing to other limbic regions and finally reaching the isocortex [24]. Structural measurements of medial temporal lobe can help to predict the progression to dementia in MCI patients [25,26,27] and asymptomatic individuals [27,28,29,30]. Hippocampal atrophy has long been considered a key early marker of Alzheimer’s disease [31,32,33]. However, hippocampal atrophy is not specific to AD and can be found in other diseases such as Lewy body dementia, vascular dementia, Parkinson’s dementia, and semantic dementia [34,35,36,37]. As a result, a single measurement of hippocampal atrophy is not sufficient to be a specific early marker of AD. Alternatively, combining the measurement of hippocampal atrophy with other brain structures that are also altered early in the course of the disease may help to improve the specificity of early biomarkers of AD [38,39].

Olfactory dysfunction is an early clinical marker of AD [40]. Olfactory deficits, such as impaired olfactory identification, were found in both AD and MCI [41,42]. The presence of an impairment in olfactory identification capacity better predicts the conversion from MCI to dementia [43,44]. Recently, it has been found that olfactory identification could be altered as early as in the SCD stage, for a meta-analysis see [45]. Olfactory identification deficit might be the consequence of damage to central olfactory structures such as olfactory bulbs (OB) and the primary olfactory cortex (POC). Central olfactory processing starts with the reception of odorant information from nasal olfactory receptors to OB. Then, the OB project to the POC (the piriform cortex, the anterior olfactory nucleus, the olfactory tubercle, the anteromedial part of the entorhinal cortex, the periamygdaloid cortex, the anterior cortical nucleus, and the nucleus of the lateral olfactory tract of the amygdala [46]. The POC, more specifically the piriform cortex, sends direct input to the lateral entorhinal cortex [47] which responds to odorant stimulation [48] and transmits olfactory information to the hippocampus [17,49]. It has been found that both OB and POC exhibit AD-related damage. OB is the first central relay in the olfactory processing pathway, and typically exhibits amyloid-β deposition and neurofibrillary tangles in patients with AD or MCI [50,51,52]. Structures of the POC and especially the entorhinal cortex were found to be atrophied in AD and MCI [21,53]. The atrophy of the POC predicts the conversion to AD [25,54,55,56] and could help with the diagnosis of MCI [57]. The atrophy of olfactory processing brain regions, such as OB and POC, could explain olfactory deficits in the course of AD. Measurement of such structures has the potential to become an early specific biomarker of AD when combined with hippocampal atrophy.

No systematic review or meta-analysis has addressed the volumetric loss of structures related to the sense of smell in the course of AD. The results of a systematic review and a meta-analysis could provide a neurobiological underpinning of the olfactory impairment that is commonly found in AD. In addition, a better characterization of the disease-specific atrophies in AD dementia and MCI stages may lead to the development of new biomarkers for the early detection of AD, which will be combined with measurements of brain structures that are altered early in the course of the disease. Thus, the aim of the study was to verify whether the presence of a clinical diagnosis of AD or MCI is associated with an atrophy of OB and/or POC compared to healthy elderly from the same age group. We hypothesized that a smaller volume of OB and/or POC could be detected in AD and MCI compared to healthy controls.

## 2. Materials and Methods

This study has been conducted following PRISMA guidelines [58]. The protocol of this study was not registered.

### 2.1. Eligibility Criteria of the Selected Studies

Eligible studies were required to meet the following criteria: (1) contain an MRI measurement of OB and/or POC volumes, (2) include a clinical group (AD dementia or MCI) and a control group of cognitive healthy participants, and (3) both title and abstract had to be written in English.

Patients from AD groups had to meet the criteria for a clinical diagnosis of AD, characterized by a significant and progressive decline in two or more cognitive domains typically lead to memory deficits, behavioral symptoms, impairment of activities of daily living, and dementia [32,59].

Patients from MCI groups had to meet the criteria for a clinical diagnosis of MCI, characterized by the presence of cognitive or memory complaints, objective cognitive impairment, and a preserved independence in functional abilities that exclude dementia [60,61]. Participants from the control groups were cognitively normal individuals.

#### Outcome

In each eligible study, total volume of both OB and /or POC had to be calculated from MRI scans by a manual or automatic segmentation from T1- or T2-weighted sequences.

### 2.2. Search Strategy and Information Source

**Figure 1:**
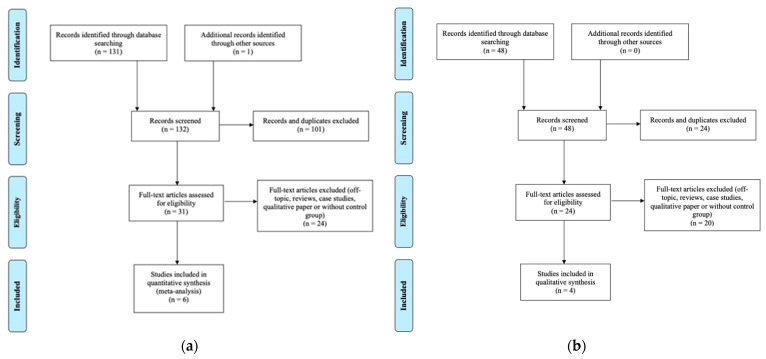
(**a**) Flowchart illustrating the selection of OB studies. (**b**) Flowchart illustrating the selection of POC studies.

We searched for studies published up to February 2021 in PubMed, PsychNet, and Ebsco databases. Unpublished theses were found using the ProQuest Dissertations and Theses database. The following keywords were used: “Alzheimer”, “mild cognitive decline”, “MCI”, “MRI”, “volum*”, “thickness”, “olfactory bulb”, “olfactory cortex” using the following syntax: (“Alzheimer” OR “mild cognitive impairment” OR “MCI”) AND (“MRI” OR “volum*” OR “thickness”) AND (“olfactory bulb” OR “olfactory cortex”). We also used the snowballing method and examined reference lists from eligible studies found in databases. After excluding duplicated studies, we reviewed 93 studies for OB comparison and 39 studies for POC comparison (See Figure 1). We then excluded reviews, case studies, qualitative papers, and off-topic studies (e.g., animal studies, no MRI data, absence of control group, etc.). As a result, 31 potentially eligible studies were identified for OB and 24 for POC.

### 2.3. Study Selection and Risk of Bias in Individual Studies

Based on the eligibility criteria mentioned above, the first author (BJ) evaluated all of the selected studies. The first author then sent the list of potentially eligible studies to a research assistant who was blind to the purpose of the study. Articles were included if they were approved by both evaluators based on the risk of biased assessment.

The risk of bias of the selected studies was assessed using the Newcastle-Ottawa Scale (NOS) [62] as recommended [63]. The NOS is a tool to evaluate the quality of non-randomized case-control studies included in meta-analyses. Criteria were based on the evaluation of participants’ selection, the comparability between groups, and the ascertainment of the quality of methods used to measure OB or POC volumes. It was agreed that the most conservative result would be selected when disagreements would emerge between both evaluators. No major disagreement emerged, and no studies were excluded following this evaluation. However, it has to be mentioned that both evaluators were unable to assess the risk of bias for two studies, as they were written in Chinese [64,65]. These two studies were included in the eligible studies, as all relevant data for the meta-analysis were present in the abstracts written in English.

### 2.4. Analysis

We used *Meta-Essential* [66] to perform analyses. We calculated Hedges’ *g* to obtain a standardized effect size for each comparison using the mean volumes and standard deviations reported in the eligible studies. When a study reported two volumes from the same structure (e.g., left and right volumes reported separately), a single effect size was calculated using the standard and recommended procedures [67] in order to avoid assigning more weight to studies with multiple outcomes. In this case, the effect size is computed as the mean of the left and right structure effect sizes:

Mean effect size=(Hedges’ g1+Hedges’ g2) / 2

The variance of this mean is:

Var (Mean effect size)= Var1+ Var2+2r√Var1√Var2

In this equation, *r* is the correlation coefficient that describes the extent to which left and right structure volume co-vary.

Then, we calculated a combined effect size when the number of studies was appropriate (≥5) [67]. We followed recent guidelines for interpreting combined effect sizes as small (*g* ≥ 0.16), medium (*g* ≥ 0.38) and, large (*g* ≥ 0.76) in geriatric populations [68]. We used the more conservative random effects model to compute the significance level of the mean effect sizes for each study.

#### Risk of Bias across Studies

We qualified the presence of heterogeneity using Cochrane’s Q-statistic and generated *I*^2^ to quantify the degree of heterogeneity among effect sizes [69]. We assumed heterogeneity if P_Q_ was significant at *p* < 0.05. When heterogeneity was assumed and the number of included studies was sufficient [70], we then tested the effect of potential moderators such as age, sex, scanner type, software used to perform analyses, MRI sequences, and the type of view (sagittal VS coronal) used.

We qualified publication bias using the Rosenthal’s failsafe-N test that gives the number of potential unpublished studies that are required to turn the combined effect size statistically insignificant or to change the conclusions of the meta-analysis [71].

## 3. Results

### 3.1. Volumetry of the OB in Patients with AD

#### 3.1.1. Study Selection and Characteristics

After analyzing full-text articles, six studies met the criteria for a total of 152 patients with AD and 166 controls (See Table 1).

#### 3.1.2. Main Effect

**Figure 2:**
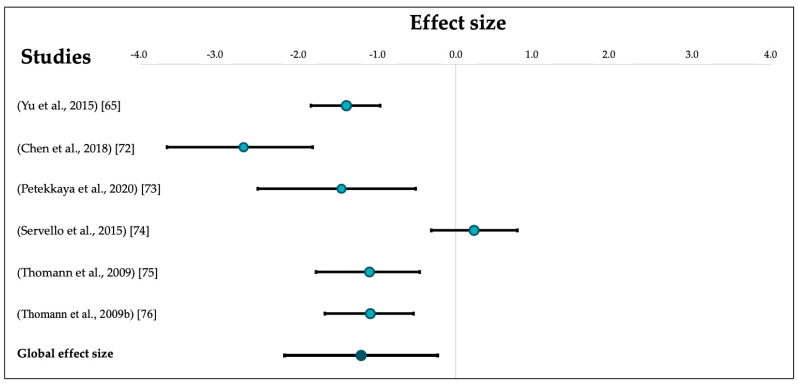
Forest plot of effects sizes for OB volumetric comparisons between patients with AD and controls. Error bars represent 95% CIs.

After combining individual effect sizes, our analysis revealed a large effect size indicating smaller OB volumes in patients with AD compared to healthy older people (k = 6, *g* = −1.21, 95% CI [−2.19, −0.24]) that was significantly heterogeneous (Q = 41.37, pQ < 0.00) (See Figure 2). Heterogeneity was confirmed by a second quantitative indicator (*I*^2^ = 87.92%).

The Rosenthal’s failsafe-N was 180 which is large and suggests no publication bias.

### 3.2. Volumetry of the OB in Patients with Mild Cognitive Impairment

#### 3.2.1. Study Selection and Characteristics

For the comparison between patients with MCI and controls, we found three different studies for a total of 104 patients with MCI and 108 controls (See Table 2).

#### 3.2.2. Effect Sizes

We did not combine different effect sizes due to the small number of studies. In two studies [63,75], participants from the MCI group exhibited smaller OB volumes compared to controls (*g* = −0.52, 95% CI [−1.05, 0.00]; *g* = −1.95, 95% CI [− 2.45, −1.49]). However, in the third study [74], MCI patients exhibited larger OB volumes than controls (*g* = 0.14, 95% CI [−0.40, 0.69]).

All studies comparing OB volume between patients and controls used well-known clinical criteria to select their participants and the majority used a manual segmentation technique to measure OB volume. One study used an automatic parcellation of OB volumes [73]. Most studies measured OB volume controlling for factors such as total intracranial volume, age, sex, and education, except for one study that did not control for these factors [74].

### 3.3. Volumetry of the Primary Olfactory Cortex

Four studies met the criteria, but two studies used the same sample, leading to a total of three eligible samples. This prevented us from carrying out a formal meta-analysis. Again, all studies comparing POC volume between patients and controls used well-known clinical criteria or a clinical rating scale, to selected their participants. One study used an automatic to segmented the POC volume and two studies used a manual segmentation method. Each study measured OB volume controlling for factors such as total intracranial volume, age, sex, or education.

A general trend for smaller structures in both AD and MCI groups compared to the control groups is observed (See Table 3). Indeed, three studies compared POC volumes between patients with AD and controls. Two studies found a significantly smaller volume in patients with AD, one study reporting a more important decrease in the left POC for those with AD [77]. Among these three studies, two studies included MCI groups. Both studies found smaller POC volumes in patients with MCI compared to controls, but only one comparison was significant [53]. One study compared patients with early MCI to those with late MCI and reported a smaller volume for the early MCI group [78].

## 4. Discussion

This meta-analysis and systematic review examined neuroanatomical structures involved in primary olfactory processing in both MCI and AD. We found a lower OB volume in both clinical groups compared to those in the control groups. When looking at the POC, despite the small number of the studies included in the present meta-analysis, a trend for lower volume is also found in both clinical groups compared to those in the control groups. These results are consistent with the hypothesis of a progressive atrophy of brain structures involved in olfactory processing in the course of AD. Volumetric reduction of olfactory brain structures is measurable as early as the MCI stage and is still more severe at the dementia stage.

The volumetric reductions in olfactory brain structures are in line with post-mortem studies that showed the presence of amyloid-β plaques and neurofibrillary tangles in both OB and POC of patients with AD [51,52,80]. Kovacs et al. (2001) demonstrated and argued that OB damage occurs very early in Braak’s staging (i.e., stage 0 or I) before AD pathology spread through the central olfactory system [80]. Our results regarding the volumetric reduction of OB and POC in patients with MCI or AD support this hypothesis. Volumetric reduction of these structures might have resulted from neurodegeneration due to the accumulation of amyloid-β plaques and neurofibrillary tangles [18]. Thus, amyloid-β plaques and neurofibrillary tangles are hypothesized to cause early damage in OB and POC of patients with AD and could result in a volumetric reduction of these structures that is measurable from MRI scans. However, it is important to note that this meta-analysis and systematic review included studies based on clinical criteria for both AD and MCI rather than on specific neuropathological measurements of AD. Therefore, future studies should include measurements of AD-pathology, such as CSF amyloid-β, amyloid PET, CSF phosphorylated tau, and tau PET, in order to verify that damages to olfactory structures are the direct expression of AD pathology. This consideration is particularly important in studies involving MCI patients since it is only a portion of MCI patients that will convert to dementia (≈34%), and more specifically, ≈31% of MCI patients that will convert to Alzheimer dementia type [8].

Neurodegeneration could explain olfactory deficits found in AD. The disease affects main olfactory functions such as odor detection threshold, discrimination of different odors, with a more severe deficit in higher-order olfactory tasks such as identification and recognition of odors [41]. One study found that left hippocampus volume reduction is related to poorer olfactory identification, which requests both olfactory and memory abilities [81]. The current study shows that the volumetric reduction observed in the course of AD is not specific to hippocampal structures and is found in other brain structures related to olfactory functions, i.e., the OB and POC. OB volume was found to be related to some specific olfactory functions such as odor identification [82] and odor detection threshold [83,84]. Thus, impaired performance of patients with AD on these functions might have resulted from neurodegeneration that occurred in OB. POC volume was also found to be related to some specific olfactory functions. One of the POC structures, the piriform cortex, is responsible for encoding odor objects [85]. Deficits in olfactory functions such as odor identification were found to be strongly correlated with tau and amyloid deposition within this structure [86,87,88]. Another structure of the POC, the entorhinal cortex, seems to be involved in olfactory functions as this structure plays a role in the transmission of olfactory information to the hippocampus [17,49]. However, the specific role of the entorhinal cortex in olfactory functions remains unclear, as very few studies have investigated this question. On a structural level, Petekkaya et al. [73] showed a significant and positive correlation between the volume of the entorhinal cortex and the OB. On a behavioral level, Devanand et al. [89] did not find any correlation between the entorhinal cortex volume and scores of odor identification. More behavioral and neuroimaging studies are needed to better understand the role of these structures and to better qualify the consequences of OB and POC volume reduction on olfactory functions in AD.

From a clinical point of view, neuroimaging techniques allow the quantification of brain structures and thus provide the possibility to detect in vivo cerebral atrophy, which can be used as a marker of neurodegeneration. Our results suggest that a volume reduction of OB and POC can be observed early in the course of the disease and can be detected from the MCI stage. Thus, OB and POC volume reduction might be new interesting biomarkers of AD. However, olfactory dysfunctions and atrophies in olfactory-related structures are not specific to AD and are also present in other neurodegenerative diseases such as Parkinson’s disease [90]. Therefore, we propose to combine OB and POC volume reduction with more traditional biomarkers such as hippocampal atrophy to enhance the specificity of the early diagnosis of AD. As a result, using this new combining approach, we might increase the detection of those with MCI that will convert to AD.

At a methodological level, although we found a global effect size in favor of an OB volume reduction of patients with AD compared to healthy older controls, it was statistically heterogeneous. When analyzing the clinical and methodological diversity among studies, they were all very similar. Since our research question was precise and because the studies included in the meta-analysis shared many similarities, we concluded that the combination of different effects sizes was appropriate. The small number of studies included (*n* = 6) prevented us from conducting moderator analysis, which is what is recommended when heterogeneous effect sizes are found [69]. Factors such as the hardware/software used, the type of scanner or sequence used to measure the OB volume could explain such heterogeneity [91,92]. OB volume is also known to have a large interindividual variability and this variability could also explain the heterogeneity [80]. Another factor that could explain heterogeneity is that not all studies controlled for total intracranial volume, which is an important covariate to take into consideration when analyzing volumetric data. Finally, heterogeneity could be explained by the fact that the majority of studies included used a manual segmentation technique instead of an automatic segmentation technique to obtained OB volumetric data. Futures studies should focus on the development of automatic segmentation methods of the OB [93].

This meta-analysis and systematic review have certain limitations. The most apparent is the small number of studies included. In fact, one of the main results of this study is that there is a lack of scientific literature for studies that have examined brain structures related to olfactory functions in the course of AD. Therefore, with only four studies resulting from the systematic review process that compared POC or OB volume between patients with MCI and healthy elderly controls, we were unable to conduct a meta-analysis using a random-effects model, as is typically recommended [94]. Regarding the selection bias of the studies included in the reviews, we were unable to evaluate the quality of two studies [63,64] as only the title and abstracts were written in English (full texts were in Chinese and we received no response from the authors). However, we decided to include these two studies in the meta-analysis, since all pertinent information was accessible in the abstracts and the studies were published in peer-reviewed journals. For all the included studies except the last two, we used the NOS tool to assess the risk of bias as recommended [62]. No studies were excluded following the risk of bias assessment. Finally, a close examination of the included studies showed some divergence on the structures included in the POC. Several models of the POC have been conceptualized [95,96,97], but they generally included common structures such as the piriform cortex, the anterior olfactory nucleus, the amygdala, the periamygdaloid cortex, and the anterior performed substance. Nevertheless, there is a need for a better classification of the structures included in the POC, especially if POC volume is used as an early biomarker of AD.

Our results indicate a volumetric reduction of both OB and POC in patients with AD and results of studies from the systematic review show that this reduction is also present in patients with MCI. New studies are needed to better characterize the degree of volume reduction of both OB and POC in patients with MCI or those that are in an earlier stage of the disease, for instance those with a SCD [5]. Second, no studies included the distinction between amnesic and non-amnesic MCI groups. Future studies should compare olfactory structures between these subgroups since amnesic MCI has been associated with a greater olfactory impairment compared to non-amnesic MCI patients [98]. Third, there is a need to encourage longitudinal studies that focus on volume reduction of olfactory-related structures in the course of AD. Results from these studies could support the hypothesis that the volume of neuroanatomical structures involved in olfactory processing decrease as the disease progresses. Longitudinal studies with larger samples of cognitively healthy participants at baseline could also lead to the analysis of the predictive value of these volumetric measurements on the development of AD-related cognitive and olfactory decline. Lastly, future researches should focus on a better characterization of the POC and on the development of fully automatized segmentation methods of these structures.

## 5. Conclusions

To conclude, a volumetric reduction of the neuroanatomical structures involved in olfactory functioning is present in patients with AD and can be measured as early as the MCI stage. Combining this neuroanatomical finding with more traditional biomarkers of AD, such as the hippocampal atrophy, volumetric reduction of OB and POC could increase the specificity of the early diagnosis of AD.

## Figures and Tables

**Table 1:** Characteristics of studies including patients with AD in the meta-analysis on OB.

Authors	Participants’ Selection	Group Comparability	OB Measurement	Sample Size	Mean Age (SD)	OB Volume (SD)
Yu et al., 2015 [65]	N/A.	N/A.	N/A.	AD: 50 Controls: 50	N/A.	AD: 30.05 (5.08) Controls: 36.46 (4.11)
Chen et al., 2018 [72]	+ NINCDS-ADRDA criteria used by two trained neurologists. + Controls were from the same community. + Consecutive recruitment. − No description of controls’ health history. − No measurement of AD-pathology biomarker (PET/CSF tau and amyloid-β)	+ Control for age, sex, education, and total intracranial volume.	+ Philips 3.0T MR scanner. + Sagittal 3D gradient-echo T1-weighted sequence. − Planimetric manual contouring. + Same method for both groups.	AD: 20 Controls: 25	AD: N/A. Controls: 55+	AD: 27.39 (3.22) Controls: 37.35 (4.04)
Petekkaya et al., 2020 [73]	+ NINCDS-ADRDA criteria. + Random recruitment of controls with equivalent for age and education level. + Controls without history of brain pathology or disease equivalent to AD or brain trauma, brain tumor, attacks, or clinical history with other accompanying psychological symptoms. − No measurement of AD-pathology biomarker (PET/CSF tau and amyloid-β).	+ Control for age and education level.	+ Philips 1.5T MR scanner. + 3D axial T1-weighted sequence. + Automatic parcellation of OB volumes using the IBASPM toolbox. + Same method for both groups.	AD: 9 Controls: 12	AD: 73.13 (4.73) Controls: 72.47 (3.35)	Left OB: AD: 0.84 (0.18) Controls: 1.04 (0.14) Right OB: AD: 0.85 (0.32) Controls: 1.21 (0.10)
Servello et al., 2015 [74]	+ NINCDS-ADRDA criteria. + Neuropsychological, radiological, and olfactory evaluation. + Controls were from the same community. + Recruitment between January and October 2013. − No random recruitment. − No description of controls’ health history. − No measurement of AD-pathology biomarker (PET/CSF tau and amyloid-β).	− No control for sex, age, or other factors.	+ Siemens 3.0T MR scanner. +T1-weighted TSE coronal plane, T2-weighted TSE coronal plane, and T2 space 3d axial plane sequences + Manual segmentation of T1 and T2-weighted coronal sections. + Same method for both groups.	AD: 25 Controls: 28	AD: 73.7 (6.8) Controls: 69.4 (9.2)	AD: 35.91 (8.90) Controls: 33.49 (11.60)
Thomann et al., 2009 [75]	+ Ascertainment of personal/family history, physical, neurological, and neuropsychological examination. + NINCDS-ADRDA criteria. + Controls from the same community. + Recruitment between 2003 and 2004. − No consecutive/random recruitment. −No measurement of AD-pathology biomarker (PET/CSF tau and amyloid-β).	+ Control for age, gender, education, and total intracranial volume.	+ Siemens 1.5-T MR scanner. + T1-weighted 3D MPRAGE sequence. + Manual segmentation. + Same method for both groups.	AD: 21 Controls: 21	AD: 71.76 (4.94) Controls: 70.38 (7.14)	AD: 83.36 (9.01) Controls: 94.52 (11.26)
Thomann et al., 2009 [76]	+ Ascertainment of personal/family history, physical, neurological, and neuropsychological examination. + NINCDS-ADRDA criteria for AD. + Controls from the same community and without cognitive complaints. + All participants were recruited between 2003 and 2004. + Controls were from the same community and without cognitive deficits. − No random recruitment. − No measurement of AD-pathology biomarker. (PET/CSF tau and amyloid-β).	+ Control for age, gender, education, and total intracranial volume.	+ Siemens 1.5-T MR scanner. + T1-weighted 3D MPRAGE sequence. + Manual segmentation. + Same method for both groups.	AD: 27 Controls: 30	AD: 71.44 (3.94) Controls: 70.50 (5.48)	AD: 85.92 (8.18) Controls: 95.73 (9.77)

Note: AD: Alzheimer’s Disease, OB: Olfactory bulb, SD: Standard deviation, N/A.: Not available. NINCDS-ADRDA: National Institute of Neurological and Communicative Disorders and Stroke and the Alzheimer’s Disease and Related Disorders Association.

**Table 2:** Characteristics of studies including patients with *MCI* in the meta-analysis on OB.

Authors	Participants’ Selection	Group Comparability	OB Measurement	Sample Size	Mean Age (SD)	OB Volume (SD)
Hang et al., 2014 [64]	N/A.	N/A.	N/A.	MCI: 50 Controls: 50	N/A.	MCI: 36.47 (4.12) Controls: 46.71 (6.25)
Servello et al., 2015 [73]	+ Petersen criteria. + Neuropsychological, radiological, and olfactory evaluation. + Controls from the same community. + Recruitment between January and October 2013. − No random recruitment. − No description of controls’ health history. − No distinction between amnesic and non-amnesic MCI. −No measurement of AD-pathology biomarker (PET/CSF tau and amyloid-β).	− No control for sex, age, or other factors.	+ Siemens 3.0T MRI scanner. +T1-weighted TSE coronal plane, T2-weighted TSE coronal plane, and T2 space 3d axial plane sequences. + Manual segmentation of T1 and T2-weighted coronal sections. + Same method for both groups.	MCI: 25 Controls: 28	MCI: 74.5 (7.5) Controls: 69.4 (9.2)	MCI: 34.87 (6.60) Controls: 33.49 (11.60)
Thomann et al., 2009 [75]	+ Ascertainment of personal and family history, physical, neurological, and neuropsychological examination. + Controls from the same community. + Recruitment between 2003 and 2004. −No measurement of AD-pathology biomarker (PET/CSF tau and amyloid-β). − The aging associated cognitive decline was considered as a conceptual equivalent for MCI. Criteria were: (1) Performance of at least one standard deviation below the age-adjusted norm on a standardized test of cognition, (2) Exclusion of any medical, neurological, or psychiatric disorder that could lead to cognitive deterioration, (3) normal activities of daily living, (4) no dementia. − No random recruitment. − No distinction between amnesic and non-amnesic MCI.	+ Control for age, gender, education, and total intracranial volume.	+ Siemens 1.5-T MR scanner. + T1-weighted 3D MPRAGE sequence. + Manual segmentation. + Same method for both groups.	MCI: 29 Controls: 30	MCI: 71.38 (6.14) Controls: 70.50 (5.48)	MCI: 90.81 (9.27) Controls: 95.73 (9.77)

Note: MCI: Mild Cognitive Impairment, OB: Olfactory bulb, SD: Standard deviation, N/A.: Not available.

**Table 3:** Characteristics of studies included in the POC systematic review.

Authors	Participants’ Selection	Group Comparability	POC Measurement	Sample Size	Mean Age (SD)	Outcome
Al-Otaibi et al., 2020 [77]	+ Diagnostic according to the National Institute on Aging—Alzheimer’s Association (NIA-AA) criteria. + MMSE to qualify controls as cognitively normal. + Participants underwent a pre-screening visit including medical history questionnaire and blood analysis. − No random recruitment. − Poor description of control’s recruitment. − No description of controls’ health history. −No measurement of AD-pathology biomarker (PET/CSF tau and amyloid-β).	+ Control for sex, age, and education.	+ Siemens 1.5 T MR scanner. + T1-weighted sequence. + Automatic segmentation using the Automatic Anatomical Labelling atlas. Targeted structures: the olfactory tract, amygdala, piriform cortex, anterior perforated substance, the subcallosal area (including the subcallosal cingulate gyrus), and the anterior cingulate cortex. − Olfactory tract is included in the definition of the olfactory cortex although it is constituted of white matter. + Same method for both groups.	AD: 14 Controls: 25	AD: 75.06 (4.60) Controls: 71.1 (5.22)	Olfactory cortex volume is significantly smaller in patients with AD compared to healthy older controls. The decrease was more apparent in the left olfactory cortex.
Lu et al., 2019 * [78,79]	+ Use of the Clinical Dementia Rating, the MMSE, the CVLT-II, the Dementia Rating Scale and a reviewed of the medical records of AD and MCI patients. + Controls were from the same community and without cognitive deficits. − No random recruitment. − Poor description of control’s recruitment. − No description of controls’ health history. − No distinction between amnesic and non-amnesic MCI. −No measurement of AD-pathology biomarker (PET/CSF tau and amyloid-β).	+ Control for age.	+ Siemens Trio 3.0 T scanner. + T1-weighted MPRAGE sequence. − Manual segmentation. Targeted structures: the anterior olfactory nucleus, olfactory tubercle, piriform cortex, anterior portion of the periamygdaloid cortex, amygdala, and anterior perforated substance. + Same method for both groups.	AD: 26 EMCI: 36 LMCI: 31 Controls: 44	AD: 71.55 (7.3) EMCI: 71.69 (7.3) LMCI: 72.41 (7.4) Controls: 74.18 (6.1)	There was a decreasing trend for a smaller POC volume dependent on AD disease state, but no difference reach significance (Controls > LMCI > EMCI > AD).
Vasavada et al., 2015 [53]	+ Diagnostics were made by a certified neurologist using NINCDS-ADRDA criteria (AD) and Peterson criteria (MCI). − Poor description of recruitment procedures. − No distinction between amnesic and non-amnesic MCI. − No measurement of AD-pathology biomarker (PET/CSF tau and amyloid-β).	+ Correction for intracranial volume and age.	+ Siemens 3.0 T MRI system. + T1-weighted MPRAGE images. − Manual segmentation. Targeted structures: the anterior olfactory nucleus, olfactory tubercle, piriform cortex, anterior portion of the periamygdaloid cortex and amygdala, and anterior perforated substance. + Same method for both groups.	AD: 15 MCI: 21 Controls: 27	AD; 71.9 (11.9) MCI: 73.2 (9) Controls: 69.5 (10.4)	MCI and AD patients had a significantly lower POC volume than controls. The difference between AD and MCI patients did not reach significance.

Note: AD: Alzheimer’s disease, LMCI: Late mild cognitive impairment, EMCI: Early mild cognitive impairment, NINCDS-ADRDA: National Institute of Neurological and Communicative Disorders and Stroke and the Alzheimer’s Disease and Related Disorders Association, MMSE: Mini-Mental State Examination, CVLT-II: The California Verbal Learning Test Two. * The data set was provided by the authors and has been used in both studies [78,79].

## Data Availability

Since this study is a meta-analysis and a systemic review of the scientific literature, the study did not report data that were not already available.
